# GPR55 and GPR119 Receptors Contribute to the Processing of Neuropathic Pain in Rats

**DOI:** 10.3390/ph15010067

**Published:** 2022-01-05

**Authors:** Ángel Zúñiga-Romero, Quetzali Rivera-Plata, Jesús Arrieta, Francisco Javier Flores-Murrieta, Juan Rodríguez-Silverio, Juan Gerardo Reyes-García, Juan Carlos Huerta-Cruz, Gustavo Ramírez-Martínez, Héctor Isaac Rocha-González

**Affiliations:** 1 Sección de Estudios de Posgrado e Investigación, Escuela Superior de Medicina, Instituto Politécnico Nacional, Miguel Hidalgo, Ciudad de México 11340, Mexico; qfb.zura@gmail.com (Á.Z.-R.); quetza.rp7@gmail.com (Q.R.-P.); jearrval@yahoo.com.mx (J.A.); fjfloresmurrieta@yahoo.com.mx (F.J.F.-M.); jrsilverio61@yahoo.com.mx (J.R.-S.); juangreyesgarcia@gmail.com (J.G.R.-G.); 2 Unidad de Investigación en Farmacología, Instituto Nacional de Enfermedades Respiratorias Ismael Cosio Villegas, Secretaría de Salud, Tlalpan, Ciudad de México 14080, Mexico; pharman007@hotmail.com (J.C.H.-C.); gramirez@iner.gob.mx (G.R.-M.)

**Keywords:** allodynia, GPR55, GPR119, neuropathic pain, spinal nerve ligation

## Abstract

Orphan G-protein-coupled receptors (GPCR) comprise a large number of receptors which are widely distributed in the nervous system and represent an opportunity to identify new molecular targets in pain medicine. GPR55 and GPR119 are two orphan GPCR receptors whose physiological function is unclear. The aim was to explore the participation of spinal GPR55 and GPR119 in the processing of neuropathic pain in rats. Mechanical allodynia was evaluated using von Frey filaments. Protein localization and modulation were measured by immunohistochemistry and western blotting, respectively. Intrathecal administration of CID16020046 (selective GPR55 antagonist) or AS1269574 (selective GPR119 agonist) produced a dose-dependent antiallodynic effect, whereas O1062 (GPR55 agonist) and G-protein antagonist peptide dose-dependently prevented the antiallodynic effect of CID16020046 and AS1269574, respectively. Both GPR55 and GPR119 receptors were expressed in spinal cord, dorsal root ganglia and sciatic nerve, but only GPR119 was downregulated after 14 days of spinal nerve ligation. Data suggest that GPR55 and GPR119 participate in the processing of neuropathic pain and could be useful targets to manage neuropathic pain disorders.

## 1. Introduction

According to the IASP, neuropathic pain is defined as a pain that arises as a direct consequence of a lesion or diseases affecting the somatosensory system. It affects 6.9% to 10% of the general population [1] and patients often experience bothersome symptoms as pain due to non-painful stimulations (allodynia) and an enhanced pain response (hyperalgesia). These symptoms frequently worsen and respond less to pharmacological treatment over time having a negative impact on quality of life [2]. To date, gabapentin, pregabalin, duloxetine, venfalaxine, and some tricyclic antidepressants have the strongest evidence for use and are recommended as first-line treatments for neuropathic pain. However, most of the drugs used are effective in less than 50% of patients and have adverse effects that restricts their medical utility [2,3]. Thus, other therapeutic options are required for patients.

Orphan G-protein-coupled receptors (GPCR) represent a diverse family of cell-surface receptors and proteins for which the endogenous ligand and function are unknown. Orphan GPCR are extensively distributed in the nervous system representing one of the most important opportunities to identify better therapeutic targets to manage neuropathic pain [4].

The orphan receptor GPR55 gene is mapped to human chromosome 2q37 which codes for a protein of 319 amino acids [5]. GPR55 receptors seem to be involved in glucose homeostasis, anti-inflammatory effects and analgesia [6,7]. In nociceptive neurons of the dorsal root ganglion (DRG), GPR55 receptor is mainly coupled to Gq and G12/13 proteins and its stimulation activates phospholipase C and RhoA GTPase to raise intracellular calcium and to inhibit voltage-gated potassium channels [8,9]. In the nervous system, GPR55 is localized in several brain regions associated with pain processing, such as the thalamus, periaqueductal gray, hippocampus, frontal cortex, striatum and brainstem, and the spinal cord [5,10,11,12]. In the DRG, it is strongly expressed in medium- to large-diameter neurons, and satellite glial cells [8,13]. The role of GPR55 in neuropathic pain remains controversial since some evidence indicates that its activation induces nociception [9,14,15], whereas other evidence shows a lack of effect [16]. Hence, the role of GPR55 in neuropathic pain processing deserves to be investigated in further experiments.

The orphan receptor GPR119 has 335 amino acids in length and is coupled to a Gs protein increasing cAMP [17,18]. This receptor has been understood to be mainly involved in glucose homeostasis and weight loss [6]. In nociception, it is highly expressed in dorsal root ganglia, trigeminal ganglia, spinal cord and some brain areas involved in pain modulation, such as the hypothalamus, hippocampus, medulla and cerebral cortex in the rat [19]. Notwithstanding this, its involvement in pain processing is unknown.

Based on the above, the current study was performed to evaluate the participation of GPR55 and GPR119 receptors in the processing of neuropathic pain induced by L5-L6 spinal nerve ligation in rats.

## 2. Results

### 2.1. Controls

**Figure 1:**
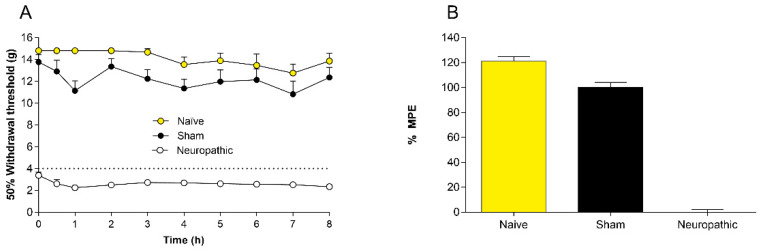
Mechanical allodynia induced by spinal nerve L5-L6 ligation. (**A**) Time courses of 50% hind paw withdrawal threshold in naïve (white circles), sham (black circles) or L5-L6 spinal nerve ligation-induced neuropathic (grey circles) rats. (**B**) Percentage of maximum effect possible (%MPE) calculated from the time courses with the different groups. Data represent the mean ± SEM of six animals per group.

L5-L6 spinal nerve ligation produced tactile allodynia in the ipsilateral left hind paw; a significant reduction in the basal 50% paw withdrawal threshold was observed in L5-L6 spinal nerve-ligated rats (3.3 ± 0.7 g) after 14 days of surgical procedure with respect to naïve rats (14.8 ± 0.5 g) or sham rats (13.8 ± 1.6 g). Moreover, the intrathecal injection of the vehicle (20 μL/rat) did not statistically modify the 50% paw withdrawal threshold of L5-L6 spinal nerve-ligated rats (2.6 ± 0.9 g), indicating that any change in the 50% paw withdrawal can be attributed to the pharmacological treatment (Figure 1). 

### 2.2. Antiallodynic Effect of Selective Antagonist GPR55 (CID16020046) and Selective Agonist GPR119 (AS1269574) in L5-L6 Spinal Nerve-Ligated Rats

**Figure 2:**
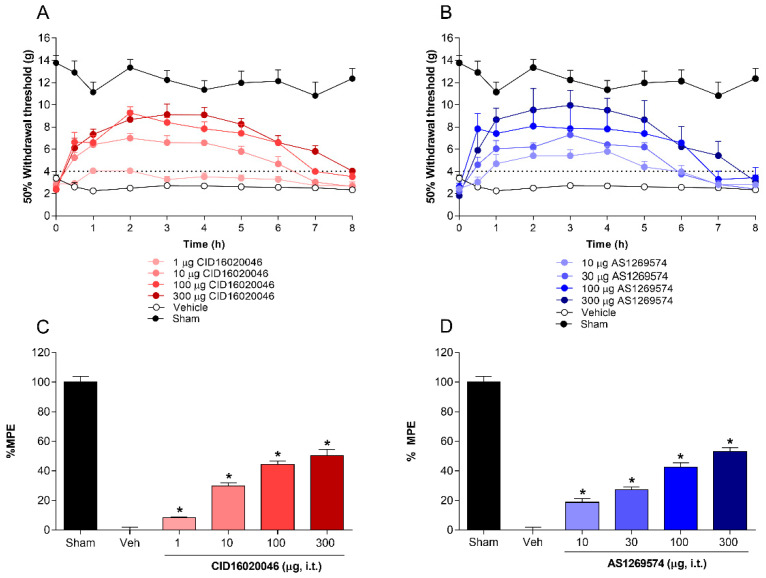
Antiallodynic effect of increasing doses of CID-16020046 (selective GPR55 antagonist; (**A**,**C**)) and AS-1269574 (selective GPR119 agonist; (**B**,**D**)) on tactile allodynia induced by L5-L6 spinal nerve ligation in rats. Data represent the mean ± SEM of six animals per group. * Significantly different from the vehicle group by one-way analysis of variance, followed by post hoc Dunnett’s test with *p* < 0.05. Abbreviations: %MPE = percentage of maximum effect possible.

The role of spinal GPR55 and GPR119 receptors in the processing of L5-L6 spinal nerve ligation-induced neuropathic pain was tested by the intrathecal injection of selective antagonist GPR55 (CID16020046) and selective agonist GPR119 (AS1269574). Intrathecal administration of CID16020046 (1–300 µg/rat. i.t.) reduced, in a dose-dependent fashion, the allodynia in L5-L6 spinal nerve ligated-rats (Figure 2A–C). Time-course data show that CID16020046 reached its maximal antiallodynic effect at around 2 h and the effect lasted for 7–8 h (Figure 2A). According to the dose–response curve, CID16020046 induced a %MPE = 50.4 ± 4.1% at 300 μg/rat (Figure 2C). In a similar way, AS1269574 (1–300 µg/rat. i.t.) diminished dose-dependently the L5-L6 spinal nerve ligation-induced mechanical allodynia (Figure 2B,D). This drug presented its maximal antiallodynic effect after about 2 h, which was sustained until 4th h, after that, it decreased gradually to basal values at 8th h (Figure 2B). In addition, AS1269574 reached an efficacy of 53.1 ± 2.7% at 300 μg/rat (Figure 2D). There were no evident adverse events at the tested doses.

### 2.3. Co-Administration of Agonist GPR55 (O-1602) or G-Protein Antagonist Peptide Reverses the Antiallodynic Effect Induced by Antagonist GPR55 (CID16020046) and Agonist GPR119 (AS1269574), Respectively, in L5-L6 Spinal Nerve-Ligated Rats

**Figure 3:**
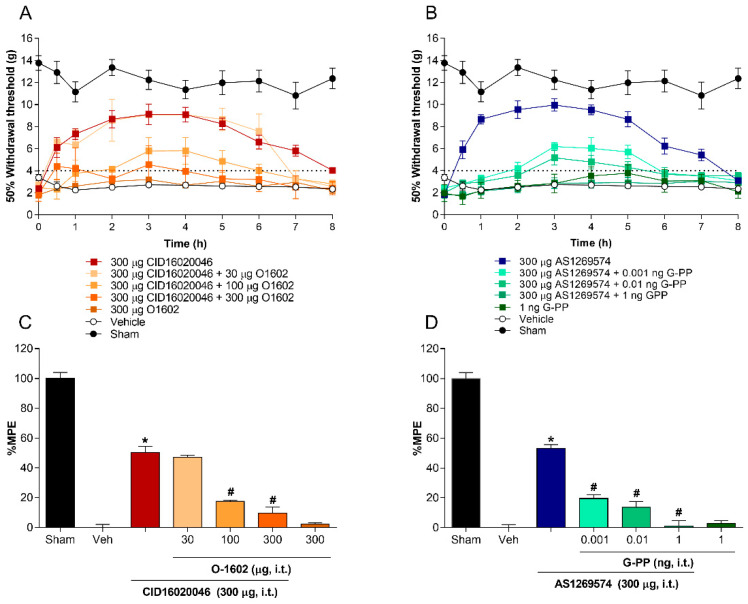
Effect of increasing doses of O-1602 (selective GPR55 agonist; (**A**,**C**)) and G-protein antagonist peptide (**B**,**D**) on antiallodynic effect induced by CID-16020046 (selective GPR55 antagonist; (**A**,**C**)) and AS-1269574 (selective GPR119 agonist; (**B**,**D**)), respectively, in L5-L6 spinal nerve ligation-induced neuropathic rats. Data represent the mean ± SEM of six animals per group. * Significantly different from the vehicle group and # significantly different from CID-16020046 or AS-1269574 group by one-way analysis of variance, followed by post hoc Dunnett’s test with *p* < 0.05. Abbreviations: %MPE = percentage of maximum effect possible.

To confirm the role of spinal GPR55 and GPR119 receptors in the processing of neuropathic pain, increasing doses of the agonist GPR55 (O1602) and the G-protein antagonist peptide were co-administered via intrathecal route with the greatest dose tested of the GPR55 antagonist CID16020046 and the GPR119 agonist AS1269574, respectively. The antiallodynic effect induced by the selective GPR55 antagonist CID16020046 (300 μg/rat, i.t.) was significantly prevented by increasing doses of the agonist O1602 (30–300 μg/rat, i.t.), as shown in Figure 3A,C. In addition, O1602 did not modify the 50% paw withdrawal threshold in L5-L6 spinal nerve-ligated rats when the drug was administered individually at the highest tested dose (300 μg/rat, i.t.). On the other hand, the reduction in the allodynia produced by intrathecal administration of the selective GPR119 agonist AS1269574 (300 μg/rat, i.t.) was reversed, in a dose-dependent manner, with increasing doses of G-protein antagonist peptide (0.001–1 ng/rat, i.t.), as shown in Figure 3B,D. Interestingly, G-protein antagonist peptide (1 ng/rat, i.t.) was not able to decrease the 50% paw withdrawal threshold when injected separately, which suggests a selective effect against the activation of spinal GPR119 receptors when co-administered with AS1269574. 

To discount the participation of systemic effects that could affect the nociceptive evaluation, plasmatic glucose levels were measured after spinal administration of CID-16020046 (300 µg), O-1602 (300 µg), AS-1269574 (300 µg) or G-PP (1 ng) at the same times that allodýnia was evaluated in the rats. Moreover, these doses were also evaluated in the rotarod-test to rule out a possible motor impairment induced by the drugs. In this regard, none of the drugs affected plasmatic glucose levels or motor coordination at the highest doses tested.

### 2.4. GPR55 or GPR119 Localization in the Spinal Cord, Dorsal Root Ganglia and Sciatic Nerve

**Figure 4:**
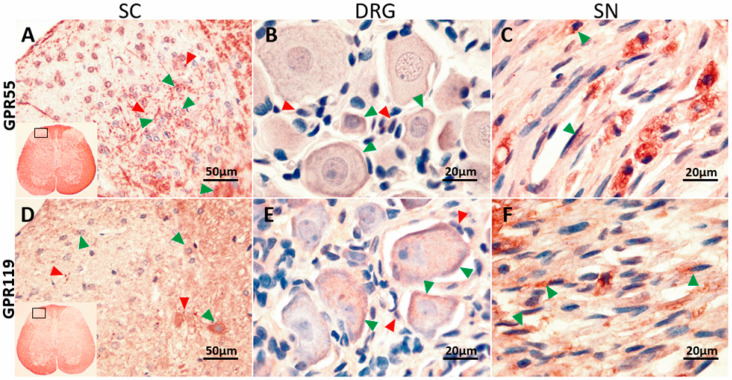
Immunolocalization of GPR55 (**A**–**C**) and GPR119 (**D**–**F**) in the left dorsal horn of the lumbar spinal cord (SC), L5-L6 dorsal root ganglia (DRG) and sciatic nerve (SN) from rats by indirect immunoperoxidase staining (labelled streptavidin biotin method). The tissue sections were counterstained with haematoxylin. Green arrowheads indicate the expression of both receptors on some neurons, whereas the red arrowheads indicate the expression on glial cells. The rectangle within small spinal cord inserts (**A**,**D**) indicate the region where the enlarged images were obtained.

To analyze the localization of both receptors in the nociceptive pathway, immunohistochemical staining of the lumbar spinal cord, left L5-L6 DRG, and left sciatic nerve was performed in neuropathic rats. GPR55 was localized in the whole spinal cord. In the white matter, it was located in glial cells, whereas in the gray matter it was found in both neurons and glial cells (Figure 4A). In the dorsal root ganglia, GPR55 was expressed in satellite cells and in small- and medium-neurons, although it was also found in large-neurons (Figure 4B). Similarly, it appeared to be present in Schwann cells in the sciatic nerve (Figure 4C). The GPR119 protein receptor had a similar expression pattern since it was also expressed in the whole spinal cord. On the dorsal horns, it was situated in glial cells and some neurons (Figure 4D), in the L5-L6 DRG, it was expressed in satellite cells and neurons (Figure 4E), whereas in the sciatic nerve, GPR119 appeared to be in Schwann cells (Figure 4F).

### 2.5. GPR55 or GPR119 Modulation in the Lumbar Ipsilateral Spinal Cord and Left Sciatic Nerve

**Figure 5:**
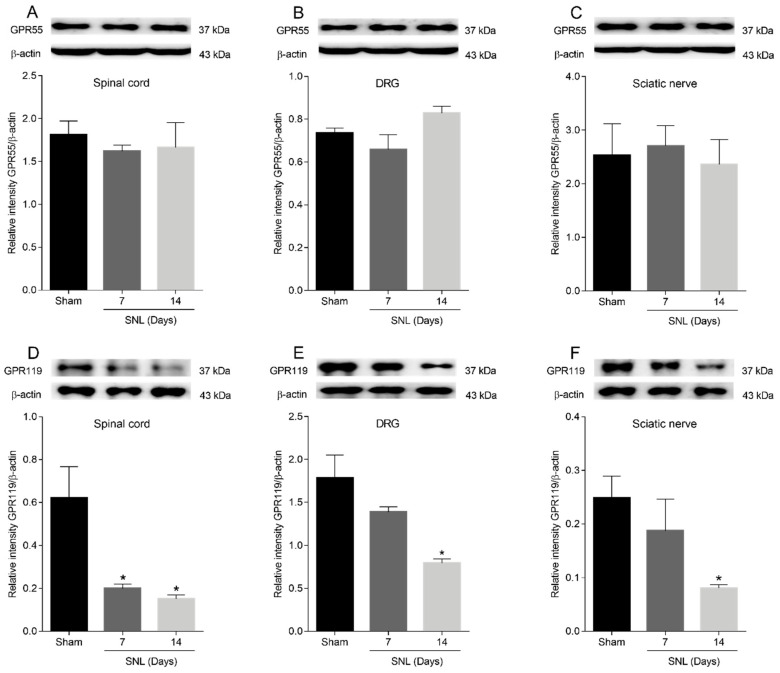
Western blotting analysis of GPR55 (**A**–**C**) and GPR119 (**D**–**F**) protein expression in ipsilateral lumbar spinal cord, L5-L6 dorsal root ganglia (DRG) and sciatic nerve of sham and L5-L6 spinal nerve ligation-induced neuropathic rats. Data were standardized in contrast to β-actin and they represent the mean ± SEM of three independent experiments per group, where each experiment incorporated the tissue mixture of three rats. * Significantly different from sham group by one-way analysis of variance, followed by post hoc Dunnett’s test with a *p* < 0.05. Abbreviations: SNL = spinal nerve ligation.

To examine the possible modulation of GPR55 or GPR119 receptors in the lumbar ipsilateral spinal cord and left sciatic nerve in L5-L6 spinal nerve-ligated rats the western blot technique was performed. In the western analysis, the bands corresponding to GPR55, GPR119 and β-actin revealed molecular weights of approximately 37, 37 and 43 KDa, respectively, in both tissues (Figure 5). Non-specific bands corresponding to GPR55 and GPR119 were absent when the membranes were incubated excluding the primary antibodies (data not shown). The GPR119 protein band decreased its expression in the lumbar ipsilateral spinal cord (Figure 5D), L5-L6 DRG (Figure 5E) and the left sciatic nerve (Figure 5F) 7 and 14 days after L5-L6 spinal nerve ligation. In contrast, the GPR55 protein band showed similar expression in the lumbar ipsilateral spinal cord (Figure 5A), L5-L6 DRG (Figure 5B) and left sciatic nerve (Figure 5C) between sham rats and L5-L6 spinal nerve-ligated rats after 7 and 14 days of the surgical procedure.

## 3. Discussion

In the current study, the results suggest that the receptor GPR55 had a pronociceptive role in neuropathic pain since CID16020046, a GPR55 antagonist, reduced tactile allodynia in L5-L6 spinal nerve ligation-induced neuropathic pain, and O-1602, a GPR55 agonist, prevented the antiallodynic effect induced by CID16020046. In this regard, O-1602 is considered an atypical cannabinoid that does not bind with high affinity to either CB1 or CB2 cannabinoid receptors [10]. Moreover, this drug has been postulated as a biased agonist of GPR18 receptors [20], but recent evidence with a selective agonist suggests that GPR18 activation induces anti-inflammatory and anti-nociceptive activities more than nociception [21]. On the other hand, CID16020046 is known as a selective GPR55 antagonist [22]. Thus, these effects seem to be mediated by GPR55 receptors instead of other receptors. To discount the participation of systemic effects or motor disturbances induced by spinal administration of CID16020046 or O-1602, the plasmatic glucose levels and motor coordination were evaluated with the highest doses tested of both drugs. Neither CID16020046 nor O-1602 at 300 µg modified the plasmatic glucose levels or motor performance of the animals suggesting that the observed changes in the nociceptive behaviour were specific and local to the site of administration. The present results are consistent with previous reports that suggest that GPR55 knockout mice have less tumour-induced mechanical hypersensitivity than control littermates [9], and failed to develop mechanical hyperalgesia in the partial nerve ligation model [14]. In contrast, one study concluded that there were no differences between GPR55 knockout mice and wild type mice in paclitaxel- or partial sciatic nerve injury-induced neuropathic pain [16]. This discrepancy could be related to the methodological tools used because the nociceptive role of GPR55 was described by a pharmacological approach [this study] and knockout mice generated by deleting GPR55 transmembrane domains [9,14], whereas the lack of effect was found in knockout mice produced by deleting exon 2 [16]. In this regard, it is well known that genetic animal models often activate compensatory mechanisms that recruit the expression of other genes to mitigate the consequences of the deletion of the gene studied. Moreover, the difference in the result might also be attributed to the animal handling, rodent characteristics (species, sex, age and body weight) and pain models used in the studies. 

Consistent with our results, a recent study found that GPR55 receptors localised in the periaqueductal gray (PAG) participate in the maintenance of sciatic-nerve-injury-induced neuropathic pain in rats. This effect seems to implicate the activation of descending facilitation pathways involving the rostral ventromedial medulla toward the spinal cord [15]. In a similar way, another study showed that the microinjection of lysophosphatidylinositol (LPI) in the PAG reduced the paw withdrawal latency in a phasic pain model, an effect prevented by ML-193 [11]. The results suggest that the nociceptive role of the GPR55 receptor at PAG level is due to its activation increasing intracellular calcium levels via P/Q-type calcium channels and inositol 1,4,5-trisphosphate (IP3) receptors, and inducing the depolarization of PAG neurons stimulating descending facilitation pathways.

Concordantly, GPR55 receptors in the dorsal root ganglion also raise intracellular calcium [8]. Studies in human embryonic kidney cells and DRG neurons show that GPR55-induced calcium increase is dependent on Gq and G12/13 proteins [8,9,23]. In this regard, the Gq protein pathway involves the activation of phospholipase C, and the subsequent opening of IP3-sensitive calcium channels and closing of voltage-gated potassium channels [8]. On the other hand, the recruitment of G12/13 by GPR55 activation promotes GTPase RhoA-mediated calcium mobilization from intracellular stores, which in turn stimulates the phosphorylation of mitogen-activated protein kinases (MAPK) and extracellular-regulated kinases (ERK) 1/2, as well as the induction of transcriptional factors, including nuclear-factor-activated T cells (NFAT), nuclear factor κβ (NFκβ), and cAMP response element-binding (CREB) proteins [8,9,23]. These transcriptional factors can translocate to the nucleus and regulate DNA transcription and gene expression. It has been reported that the activation of GPR55 receptors up-regulates mediators in neuropathic pain, such as interleukin (IL)-12, tumour necrosis factor (TNF)-α, interferon (IFN)-ɣ and granzyme B, among other proteins [24], and seems to regulate neuroprotective interleukins, such as IL-4 and IL-10 [14]. Interestingly, the GPR55 antagonist CID16020046 used in this study is associated with a reduction in phosphorylation of ERK1/2 [25], whereas the GPR55 agonist O-1602 increases intracellular calcium and ERK1/2 phosphorylation [20], which is in line with the signalling pathway reported for GPR55. It is worth emphasizing that the signalling mechanisms described for GPR55 here facilitate neuropathic pain processing. 

In addition, the LPI-mediated activation of GPR55 receptors increases the firing rate of low-threshold Aβ and nociceptive Aδ primary afferent fibres to mechanical stimuli [9]. In this regard, both types of fibres have been implicated in mechanical hyperalgesia, tactile allodynia and ectopic spontaneous activity since they are able to trigger and maintain an increased excitability of the spinal cord in neuropathic pain [26]. The administration of CID16020046 in the anterior cingulate cortex reduces the formalin-induced c-fos marker of neuronal activity in the spinal cord [25].

The localization and regulation of the GPR55 receptors also seems to support their nociceptive role. In our study, the GPR55 receptors were expressed in neurons of the dorsal horn of the spinal cord and dorsal root ganglia, as well as in glial cells of the spinal cord, satellite cells of the dorsal root ganglia and Schwann cells on the sciatic nerve, and this expression was not altered between naïve and neuropathic rats. Correspondingly, other studies have confirmed that GPR55 receptors are expressed along the nociceptive pathway. Thus, at the peripheral level, GPR55 receptors are expressed in DRG neurons ([8,13], this study), as well as in satellite glial cells ([13], this study) and Schwann cells [this study], two types of glia cells found in the peripheral nervous system whose activation after nerve injury participates in the development of neuropathic pain [27,28]. In the central nervous system, GPR55 is also found in important areas for pain modulation, such as the thalamus, periaqueductal gray and brainstem, among others [5,10,11,12]. In addition, it has been reported in different pain types that GPR55 receptors increase nociception in the periaqueductal gray [11,15], anterior cingulate cortex [25], rostral ventromedial medulla [15] and spinal cord [this study]. To date, all studies indicate that GPR55 receptors up-regulate [12,15,29] or maintain their expression after some noxious stimuli ([30], this study). Taken together the data suggests that the receptors are necessary for the development and maintenance of neuropathic pain. Moreover, our results confirm pharmacologically the nociceptive role of GPR55 receptors in the maintenance of neuropathic pain, show their localization for the first time in Schwann cells and spinal cord, and evaluate their participation at spinal level.

On the other hand, in the current study, the intrathecal administration of AS1269574, a GPR119 receptor agonist, produced an antiallodynic effect in L5-L6 spinal nerve ligation-submitted neuropathic rats. In line with our results, a study found that oleoylethanolamide decreases acetic acid- and formalin-induced pain through perosixome-proliferator-activated receptors (PPAR), an α-activation independent mechanism [31]. Although, the study might suggest the participation of GPR119 receptors in inflammatory and visceral pain, data should be interpreted with care because oleoylethanolamide could also act on TRPV1 [32], NMDA [31] and GPR55 [10] receptors. To date, there are no specific antagonists of GPR119, so we decided to administer a G-protein antagonist peptide (G-PP), which blocks the activation of G proteins by receptors in a reversible and competitive way [33]. In our study, G-PP was able to reverse the antiallodynic effect induced by AS1269574, at doses ineffective by themselves. Even though it has been reported that AS1269574 activates TRPA1 channels to stimulate glucagon-like peptide-1 in an independent manner to GPR119 activation [34], the antiallodynic effect of AS1269574 in this study seems to involve spinal GPR119 receptor activation since this effect was reversed by a G-protein antagonist and TRPA1 activation induces allodynia in nociceptive neurons [35]. Consistent with the behavioural results, we showed that GPR119 receptors are located in both neurons and glial cells of the spinal cord. Furthermore, they were expressed in DRG neurons, satellite cells and Schwann cells at the peripheral level, indicating their expression along the nociceptive pathway. In addition, the anti-neuropathic effect of GPR119 receptors in the spinal cord seems to be specific since spinal administration of AS1269574 (300 µg) or G-PP (1 ng) did not modify the plasmatic glucose levels or motor performance at the highest dose tested. 

Regarding their transduction mechanism, GPR119 receptors appear to be associated preferentially to Gαs proteins and elevation of cyclic adenosine monophosphate (cAMP) in transfected cells [36,37], but they have been linked to Gαi and Gαq proteins as well [37]. Another group found that GPR119 agonists differ from one another in the G proteins activated [38], an observation that could be tissue specific since the coupling to G proteins for GPR55 receptors has only been studied using cell lines. According to our results, GPR119 receptors could be coupled to G proteins at spinal level because their stimulation hyperpolarizes neurons by opening of potassium channels and closing of calcium channels [39]. Accordingly, it has been reported that the administration of AS1269574, the GPR119 receptor agonist used in this study, inhibits the phosphorylation of IκBα, JNK, and ERK [40].

Unlike GPR55 receptors, in our study, GPR119 receptors were downregulated in the spinal cord and sciatic nerve in rats submitted to spinal nerve injury. In line with our results, GPR119 transcripts are diminished in patients with intestinal inflammation [41]. Thus, the decrease of the GPR119 receptor expression in the face of noxious stimuli appears to be a regulatory mechanism that facilitates pain sensitization. In support of this view, it has been reported that some agonists desensitize GPR119 receptors after their activation [42]. This effect seems to be associated with the recruitment of β-arrestin [37], a small protein involved in the desensitization, internalization and recycling of G-protein-coupled receptors. To the best of our knowledge, this is the first study to evaluate the participation of GPR119 receptors in pain.

## 4. Materials and Methods

### 4.1. Animals

Experimental procedures were implemented in male Wistar rats weighing between 160–180 g from own breeding facilities (Animal facilities of INER, SSA Mexico City, México). Rats were maintained in an air-conditioned room at 23–25 °C on a 12-h light/dark cycle, with ad libitum access to food and water. The number of rats used was the minimal necessary to reach significant statistical power. Rats were submitted to euthanasia at the end of the experimental paradigm in a chamber with carbon dioxide.

### 4.2. Drugs

2-[[2-(4-Bromophenyl)-6-methyl-4-pyrimidinyl]amino]ethanol (AS1269574), 5-Methyl-4-[(1R,6R)-3-methyl-6-(1-methylethenyl)-2-cyclohexen-1-yl]-1,3-benzenediol (O1602), 4-[4,6-Dihydro-4-(3-hydroxyphenyl)-3-(4-methylphenyl)-6-oxopyrrolo[3,4-c]pyrazol-5(1H)-yl]benzoic acid (CID16020046) and G-protein antagonist peptide were purchased from Tocris Bioscience (Bristol, UK). All drugs were freshly suspended before each experiment in normal saline solution containing 10% of Tween 20. Drugs were selected based on their commercial availability as well as for their selectivity for GPR55 and GPR119 receptors. AS1269574 is a selective GPR119 agonist with a pEC50 = 5.6 [43], O1602 is a GPR55 agonist with a pEC50 = 7.9–8.9 [44], CID16020046 is a selective GPR55 antagonist with a pA2 = 7.3 [22] and G-protein antagonist peptide is a blocker of the activation of G proteins by receptors [33]. The drug doses were chosen from pilot experiments under our conditions.

#### 4.2.1. Induction of Spinal Nerve Ligation and Measurement of Tactile Allodynia

Rats were prepared according to the method previously described [45]. In brief, animals were anesthetized by an intraperitoneal injection of ketamine (50 mg/kg)/xylazine (20 mg/kg) combination. After surgical preparation, the dorsal vertebral column was exposed and the left L5-L6 spinal nerves were isolated and firmly tied with a 6–0 non-absorbable silk suture proximal to the dorsal root ganglion. For sham rats, the surgical procedure was similar in every detail, but the L5-L6 spinal nerves were not ligated after exposure. 

Fourteen days after surgical procedure, the rats were placed in elevated, clear-acrylic, wire mesh-bottomed chambers. After a 30 min period of acclimatization, tactile allodynia was tested using a series of standardized von Frey fibres with logarithmically incremental stiffness (Stoelting, Wood Dale, IL, USA) according to the paradigm previously described [46]. In each evaluation, a 2 g von Frey fibre was applied perpendicular to the plantar surface of the left hind paw with enough force to produce a buckle during 5 s. A positive response was registered when the rat abruptly withdrew its paw and indicated the use of the previous fibre in the series, whereas a negative response was recorded in the absence of paw withdrawal response after 5 s and prompted testing of the next fibre in the series. This procedure continued until four additional measurements were obtained after the first response change in the paw withdrawal behaviour or until five consecutive negative (15 g) or four consecutive positive (0.25 g) responses were obtained. The resulting pattern allowed the calculation of the 50% paw withdrawal threshold by the following equation.

$$50\%\;g\;\mathrm{threshold}=\frac{10^{\left(\mathrm{Xf}+\;\kappa \delta \right)}}{10,000}\;$$

where Xf = the logarithmic value of the last von Frey fibre utilized, κ = the value obtained in table for the response pattern, and ∂ = the mean logarithmic difference between stimuli. All rats were evaluated for 50% paw withdrawal threshold before drug administration and 0.5, 1, 2, 3, 4, 5, 6, 7, and 8 h after drug administration. Tactile allodynia was present when the 50% paw withdrawal threshold was below 4 g. 

#### 4.2.2. Intrathecal Administration

For the spinal drug administration, rats were anesthetized with 2% isoflurane in oxygen via nose cone. Then, the lumbar region was shaved and prepared with Betadine solution, and the intervertebral spaces were widened by placing the animal over a plexiglas tube. Rats were injected at the L5-L6 interspace using a 0.5-inch 30-gauge needle. Correct subarachnoid positioning of the tip of the needle was verified by a tail- or paw-flick reflex. Animals were recovered in the clear-acrylic, wire mesh-bottomed chambers before tactile allodynia testing [47].

### 4.3. Immunohistochemistry

Rats were euthanized with CO_2_ and perfused intra-aortically with 300 mL of ice-cold, oxygenated artificial cerebrospinal fluid (ACSF) [containing (mM): NaCl 125, KCl 2.5, CaCl_2_ 2.5, MgCl_2_ 2, NaHCO_3_ 26, NaH_2_PO_4_ 1.25, and glucose 25], pH 7.4, at a flow rate of 18–25 mL/min. Animals were decapitated, the spinal cord obtained by hydro-extrusion with ice-cold ACSF and the ipsilateral L5-L6 DRG were excised [48]. The lumbar section and the DRGs were fixated in formaldehyde during 24 h, after that, they were dehydrated through 70%, 80% 90% and three times 100% ethanol and twice xylol for 10 min each, using a tissue processor (Thermo Scientific™ Citadel 2000, MA, USA). Sections were cut from frozen blocks with a cryostat at a thickness of 30 μm for the spinal cord and 12 μm for the DRGs. Then, biopsies were embedded twice in paraffin for 1.5 h each (Shandon Histocentre 2, MA, USA). 

For immunodetection, tissues were rehydrated and then blocked with 3% H_2_O_2_ in methanol for 30 min followed by antigen retrieval performed with citrate buffer 10 mM pH 6.0 for 5 min in microwave. Slides were incubated with anti-GPR119 antibody (1:200; Bioss Antibodies, Woburn, MA, USA) and anti-GPR55 (1:100; Biorbyt, Cambridge, UK) at 4 °C overnight and then with a secondary biotinylated anti-immunoglobulin antibody followed by horseradish peroxidase-conjugated streptavidin (BioGenex, San Ramon, CA, USA). These were used according to the manufacturer’s instructions, using 3-amino-9-ethyl-carbazole (AEC, BioGenex) as a substrate in acetate buffer containing 0.05% H_2_O_2_. The tissue sections were counterstained with haematoxylin. The primary antibody was replaced by non-immune serum for negative controls. The tissues were observed under a microscope (Leica DM750, Leica Biosystems, Wetzlar, Germany). Images were captured with a 5 MP camera (Leica ICC50 E, Leica Biosystems, Wetzlar, Germany) and analysed by the software Image-Pro Premier 9.1 (Media Cybernetics, Rockville, MD, USA).

### 4.4. Western Blot Analysis

Western blot analysis was used to detect changes in the expression of GPR55 or GPR119 receptors in the ipsilateral lumbar dorsal spinal cord and sciatic nerve of rats subjected to L5-L6 spinal nerve injury. For this purpose, sham rats and L5-L6 spinal nerve-ligated rats (7 and 14 days) were submitted to euthanasia by decapitation. After, the lumbar region of the spinal cord and the left sciatic nerve were excised, and the lumbar spinal cord was dissected to isolate the ipsilateral dorsal spinal cord section. Immediately, tissues were blended in ice-cold lysis buffer (containing: 150 mM NaCl, 50 mM Tris–HCl, 5 mM EDTA, 1 mM phenylmethylsulfonyl fluoride, 10 μg/mL aprotinin, 10 μg/mL leupeptin, 10 μg/mL pepstatin A and 0.1% TritonX-100) at pH 7.4. Then, the homogenates were centrifuged at 14,000 rpm for 10 min to remove the cellular rubbish. The resultant supernatant was used to measure protein concentration by the Quick Start™ Bradford Protein Assay (Bio-Rad, Hercules, CA, USA). Fifty micrograms of protein from each sample were loaded into wells and they were resolved by 10% SDS–polyacrylamide gel electrophoresis. Next, proteins were transferred to polyvinylidene fluoride membranes and the membranes were blocked with 5% non-fat milk in ice-cold phosphate buffered saline with Tween 20 (containing: 137 mM NaCl, 2.7 mM KCl, 10 mM Na_2_HPO_4_, 2 mM KH_2_PO_4_ and 0.1% Tween 20) at pH 7.4 for 1 h. After that, membranes were incubated with the primary GPR55 antibody (rabbit anti-GPR55, 1:2000, ab203663, Abcam, Waltham, MA, USA) or with the primary GPR119 antibody (rabbit anti-GPR119, 1:5000, bs-12016R, Bioss) at 4 °C overnight. Membranes were washed three times with gentle agitation for 5 min each in ice-cold phosphate buffered saline with Tween 20. Later, horseradish peroxidase-conjugated secondary antibody (donkey anti-rabbit, 711-035-152, 1:10,000, Jackson ImmunoResearch, West Grove, PA, USA) was added to the membranes at room temperature for 1 h to recognize the primary antibody. Protein signal detection was achieved with the ECL chemiluminescence system (ECL plus, Amersham, UK). The membranes were stripped and incubated with the primary β-actin antibody (mouse anti-actin, GT5512, 1:10,000, Genetex, Irvine, CA, USA) and its respective horseradish peroxidase-conjugated secondary antibody (anti-mouse, 115-035-003, 1:10,000, Jackson ImmunoResearch Laboratories Inc., West Grove, PA, USA), which was employed as loading control to normalize GPR55 and GPR119 protein expressions. The densitometry analysis of the bands was carried out using ImageLab 5.0 software (Bio-Rad, Hercules, CA, USA). 

### 4.5. Experimental Design

The participation of spinal GPR55 and GPR119 receptors in the modulation of neuropathic pain was evaluated 14 days after spinal nerve ligation. To guarantee that all L5-L6 spinal nerve-ligated rats included in the study had tactile allodynia, the 50% paw withdrawal threshold was measured before drug administration. To determine the role of the spinal GPR55 and GPR119 receptors in the modulation of neuropathic pain, L5-L6 spinal nerve-ligated rats were administered with vehicle or increasing doses of the selective GPR55 receptor antagonist CID16020046 (1–300 µg/rat. i.t.) or the selective GPR119 receptor agonist AS1269574 (1–300 µg/rat, i.t.). To confirm pharmacologically the role of spinal GPR55 and GPR119 receptors in the modulation of neuropathic pain, increasing doses of the GPR55 agonist O1602 (30–300 µg/rat. i.t.) or the G-protein antagonist peptide (0.001–1 ng/rat, i.t.) were co-administered with the highest tested dose (300 µg/rat. i.t) of the GPR55 receptor antagonist CID16020046 or the GPR119 receptor agonist AS1269574, respectively. G-protein antagonist peptide was used to reverse the antiallodynic effect induced by the GPR119 receptor agonist AS1269574 because a selective and/or potent GPR119 antagonist is not yet available. In addition, the highest doses of O1602 (300 µg/rat. i.t.) and G-protein antagonist peptide (1 ng/rat, i.t.) were tested individually in order to discount an effect themselves on nociceptive behaviour at the co-administered doses. To discount possible systemic effects or motor impairments that could alter the nociceptive response, plasmatic glucose levels and motor performance [49] were measured after spinal administration of the drugs at the highest dose tested and at the same times that nociceptive behaviour was evaluated in the rats. The evaluator was blinded of the treatment administered to each animal. To measure the spinal expression and possible modulation of GPR55 and GPR119, we evaluated the protein expression at the lumbar spinal cord, L5-L6 DRG and left sciatic nerve by immunohistochemistry in neuropathic rats (14 days after L5-L6 spinal nerve ligation) and analysed their modulation at the ipsilateral dorsal spinal cord and sciatic nerve by western blotting in sham and neuropathic rats (7 and 14 days after L5-L6 spinal nerve ligation).

### 4.6. Data Analysis and Statistics

Behavioural data were presented as the mean ± SEM for six animals per group. Time-courses were built by plotting the 50% paw withdrawal threshold against time. An increase in the 50% paw withdrawal threshold was considered as antiallodynic effect. The area under the curve was calculated by the trapezoidal method from time-courses. Percentage of maximum effect possible (%MPE) to construct the dose-response curves was obtained using the formula:

$$\%\mathrm{MPE}=\frac{{\mathrm{AUC}}_{\mathrm{Drug}}-{\;\mathrm{AUC}}_{\mathrm{Vehicle}}}{{\mathrm{AUC}\;}_{\mathrm{Sham}}-{\;\mathrm{AUC}}_{\mathrm{Vehicle}}}\times 100$$

For protein expression, all results are given in bar plots as the mean ± SEM for 3 independent experiments, where each experiment incorporates the tissue mixture of three rats. Behavioural and molecular bar plots were analysed by one-way analysis of variance, followed by the Dunnett’s test, to compare differences between groups. The differences reached statistical significance when *p* was less than 0.05.

## 5. Conclusions

In summary, our results suggest that GPR55 and GPR119 receptors are expressed through the nociceptive pathway and they participate in the modulation of neuropathic pain. Moreover, the data appear to indicate that spinal GPR55 receptor antagonism or spinal GPR119 receptor activation could be useful strategies in neuropathic pain management.

## Data Availability

Data is contained within the article.
